# Hand Motion Capture from a 3D Leap Motion Controller for a Musculoskeletal Dynamic Simulation

**DOI:** 10.3390/s21041199

**Published:** 2021-02-08

**Authors:** Robin Fonk, Sean Schneeweiss, Ulrich Simon, Lucas Engelhardt

**Affiliations:** Scientific Computing Centre Ulm (UZWR), Ulm University, 89081 Ulm, Germany; robin.fonk@gmx.de (R.F.); publication@seanschneeweiss.de (S.S.); ulrich.simon@uni-ulm.de (U.S.)

**Keywords:** musculoskeletal hand model, hand motion, leap motion controller, motion capture, range of motion, anybody modeling system, AMS, bvh

## Abstract

The AnyBody Modeling System™ (AMS) is a musculoskeletal software simulation solution using inverse dynamics analysis. It enables the determination of muscle and joint forces for a given bodily motion. The recording of the individual movement and the transfer into the AMS is a complex and protracted process. Researches indicated that the contactless, visual Leap Motion Controller (LMC) provides clinically meaningful motion data for hand tracking. Therefore, the aim of this study was to integrate the LMC hand motion data into the AMS in order to improve the process of recording a hand movement. A Python-based interface between the LMC and the AMS, termed ROSE Motion, was developed. This solution records and saves the data of the movement as Biovision Hierarchy (BVH) data and AnyScript vector files that are imported into the AMS simulation. Setting simulation parameters, initiating the calculation automatically, and fetching results is implemented by using the AnyPyTools library from AnyBody. The proposed tool offers a rapid and easy-to-use recording solution for elbow, hand, and finger movements. Features include animation, cutting/editing, exporting the motion, and remote controlling the AMS for the analysis and presentation of musculoskeletal simulation results. Comparing the motion tracking results with previous studies, covering problems when using the LMC limit the correctness of the motion data. However, fast experimental setup and intuitive and rapid motion data editing strengthen the use of marker less systems as the herein presented compared to marker based motion capturing.

## 1. Introduction

Joint reaction forces, moments, as well as muscle activities are crucial parameters for medical implant design, rehabilitation, or biomechanical research questions. To address those musculoskeletal questions, the inverse dynamics modeling approach is an increasingly applied method. In contrast to implanted sensors for experimental measurements, this simulation approach is ethically not questionable and adaptable for parameter studies. The work of Engelhardt and Melzner et al. [1] introduced a musculoskeletal hand model developed for inverse-dynamics simulations with the AnyBody Modeling System™ (AMS). This was used to further analyze the resulting human hand joint and muscle forces in an ongoing motion sequence. The work of Rasmussen et al. [2] described the computational procedure for using the AMS. As indicated, movements of the body model are based on kinematic measurements [2]. The kinematic data are frequently recorded by using a marker-based camera motion capture system (MoCap). MoCap can be time-consuming, cost-intensive, and possibly inaccurate because tracked markers attached to the skin move relative to the bone [3]. A motion capture tracking system can take an enormous effort for the experimental setup. Applying non-anatomical parameters as video tracking markers to the model is overall a complex task. As many as 39 reflective markers have to be placed on the subject’s hand, and the camera system needs to be manually calibrated and set up. The data have to be edited and optimized to be able to transfer the motion data to the AMS skeleton. In Gragg et al. [4], this is described as having a Cartesian space on the motion capture system and transferring the positions into joint space. Alternatively, physiological joint angles could be directly applied for driving the human model. Therefore, similar to the posture reconstruction method proposed by Gragg et al., marker-less motion capture systems could be used to apply motion to body models. For gait analysis or full body modeling the Microsoft Kinect Sensor (Microsoft Corp., Redmond, WA, USA) has been used [3] and for an accurate motion capture of the hand, the Leap Motion Controller (LMC) (Leap Motion, San Francisco, CA, USA) has been identified to suit the needs for medical applications [5,6,7]. Studies [8,9,10,11,12,13] have measured the accuracy of the motion tracking of the hand with the LMC. The conclusion of Smeragliuolo et al. [12] indicated that the LMC provides clinically meaningful data for wrist flexion/extension, mostly meaningful data for wrist deviation, and inaccurate measurements of forearm pronation/supination. Chophuk et al. [11] for finger joint angles compared the LMC and a finger goniometer, with the results displaying a mean error of 5.73 degree. The finger goniometer might be the more accurate reference measurement but unable to track motions of the entire forearm in the desired way. The LMC provides the positions and orientation of the bones and joints of the entire recorded hand. Based on these values, joint drivers in the AMS can be applied to reproduce the same posture and movement. This is done by driving joint angles and therefore rotating the attached bones as part of the kinematic analysis in the AMS. The created motion is then used for the inverse-dynamic simulation that calculates musculoskeletal forces and moments.

The aim of the present work was to engineer an automatic interface between an LMC and the AMS to capture hand movements and integrate them into an inverse-dynamic musculoskeletal simulation. It should be investigated, if a sensor—mainly developed for media and entertainment purpose—is able to be used as a motion input for musculoskeletal models in biomechanical research. Limitations due to covering problems as reported by Rühlicke [14] were analyzed, proposing an accuracy measurement using video tracking in comparison to the marker-less motion capture recording. To evaluate how precise the LMC works within this new framework, the motion was compared between the video tracking and the LMC recording.

## 2. Methods

### 2.1. General Approach

**Figure 1:**
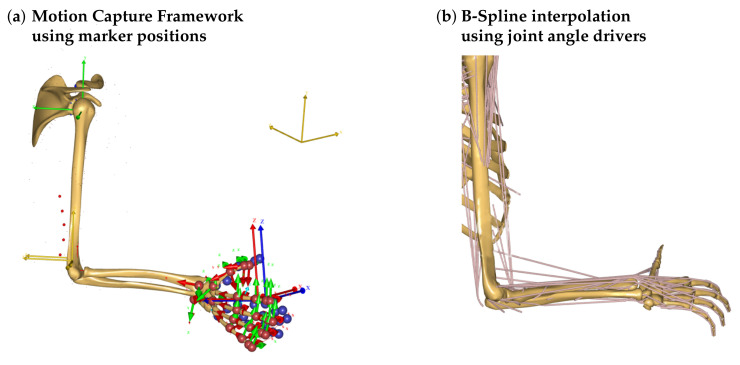
Possibilities to process recorded motion in the AMS. (**a**) MoCap simulation uses motion files with trajectories of marker coordinates. The distances between the recorded coordinates of the blue points from the movement file and the red reference points on the skeleton are minimized by solving an optimization problem. The arrows represent the orientation of the local and global coordinate systems. (**b**) Joint angle interpolation is based on B-spline interpolation of recorded motion and uses the resulting continuous function for driving the joint according to flexion/extension, abduction/adduction, and pronation/supination.

This work explains the developed tool set ROSE Motion, featuring an automated workflow from a motion recording with the LMC, generation of motion files, and import into the AMS with a subsequent kinematic analysis. This interface software connects the LMC with AMS using Python [15] and core features of the Python libraries Gooey [16] (graphical user interface for settings), AnyPyTools [17] (interface for AMS), LeapCxx [18] (interface for the LMC), and PyMO [19] (motion data handling and animation). The source code and the required Python packages are available in [20]. The software solution ROSE Motion controls the LMC. ROSE Motion is a Python program to save motion data of recorded positions, angles, finger lengths, and bone segments. The AMS uses the motion data for driving finger joint movement. This work compares two approaches to process the recorded motion within AMS:

- Marker-based C3D (three-dimensional time-sequence data) and Biovision Hierarchy (BVH) files for MoCap simulation:
  Imported trajectories of marker coordinates are fitted to model attached points using the AnyMoCap™ Framework [21]. An optimization algorithm calculates the motion of the arm while maintaining the minimum distance to the linked markers (Figure 1a) possible. This procedure requires manual adaptions of the initial position, marker alignment, and mapping prior to the simulation.
- Joint angle interpolation:
  For each joint (i.e., wrist) and each degree of freedom (flexion/extension, abduction/adduction, and pronation/supination) a (time)series of recorded angles is interpolated with a B-spline and the resulting continuous function is used to drive the respective joint. For that, the recorded motion has to be converted into subsequent joint angles and transferred into the AMS study. A neutral-zero position of the arm is used as a reference position to which all following positions are compared. The calculated joint angles will therefore describe the motion to drive the joints from the initial position to the recorded position (Figure 1b).

### 2.2. Workflow

**Figure 2:**
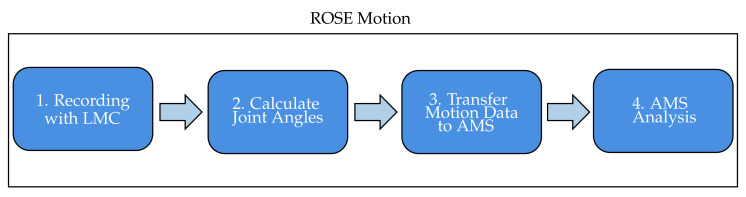
Workflow of the developed tool ROSE Motion. After the hand movement is recorded, the cardan angles of the various finger joints are calculated for each frame. The calculated time-sequence data is transferred via data files to the AMS. The simulation starts automatically.

The Python based ROSE Motion software implements the following steps (Figure 2): First, the LMC records the hand movement. Meanwhile, the software stores time-sequence data. Following termination of the recording, the Cardan angles in each joint are internally calculated. The angle values and recording information are exported to AMS data files. ROSE Motion automatically initiates the AMS simulation, and dumps the selected results for postprocessing.

#### 2.2.1. Recording with the LMC

**Figure 3:**
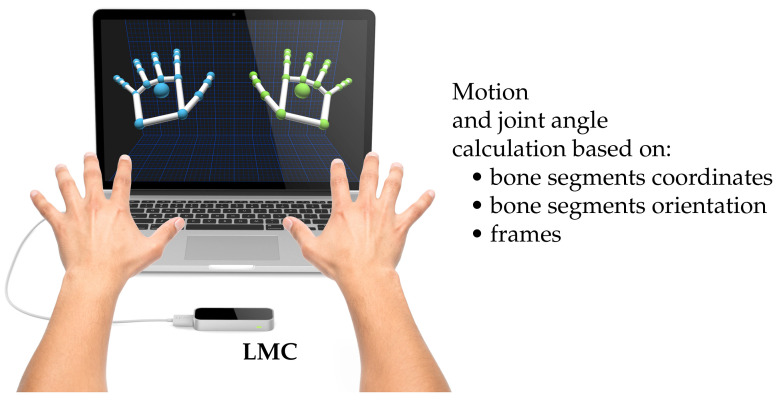
Recording the hand motion with a LMC. The LMC software visualizes the finger bones and joints of the internal hand model—marked on the screen as green and blue dots. The software ROSE Motion processes the LMC data and saves frames with motion data while recording, e.g., bone segments coordinates and bone segments orientation (source of image: the work in [22]).

In the first step, the LMC recognizes the motion of a single or both hands and ROSE Motion stores the positions and basis of each bone segment from elbow to finger tip (green and blue dots in Figure 3) according to the LMC internal hand model. The joint angle calculation is based on recorded frames, each frame holding the position and the basis of the bone segments in-between the joints.

#### 2.2.2. Calculate Joint Angles

**Figure 4:**
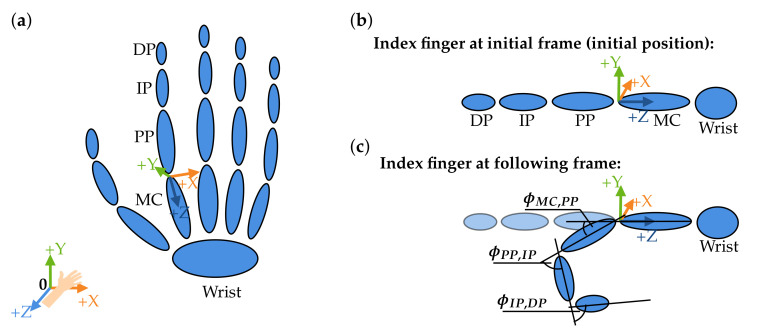
Definition of the joint angles. (**a**) The skeletal structure of a hand. (**b**) The stretched position of the index finger (wrist, metacarpals (MC), proximal phalanges (PP), intermediate phalanges (IP), and distal phalanges (DP)) represents the reference position. (**c**) Angles are calculated in a followed bending position of the index finger. The angles $\phi_{MC,PP}$, $\phi_{PP,IP}$ and $\phi_{IP,DP}$ represent the angles around the X-axis (flexion/extension).

To transfer the motion into the AMS, the corresponding angles must be calculated for each recorded frame/time step and for each joint. Accordingly, three joint angles (Cardan angles) were exported per joint, each describing a relative angle between two bone segments, i.e., metacarpal to proximal phalanx, along a selected axis [23]. In this work, the joint coordinate system followed the standards of the International Society of Biomechanics as described by Disselhorst-Klug et al. [24]. The three angles (ϕ,θ,ψ) and the corresponding axes in Figure 4 are

- rotation about the flexion and extension axis (*X*-axis, ϕ),
- rotation about the resultant abduction and adduction axis (*Y*-axis, θ), and
- rotation about the resultant rotation axis (*Z*-axis, ψ).

An initial position (neutral-zero position) of the hand is set in the AMS. The joint angle calculation is based on the reference position and bone bases of the flat outstretched hand exported from the AMS. The angles for the following motion are calculated depending on this reference position. ROSE Motion saves the basis for each bone—per frame/time step—from the LMC recording. From this basis, the required joint angles (ϕ,θ,ψ) are calculated by matrix multiplications and angle functions. As an example, the index finger is shown in Figure 4. The index finger is shown in its initial position and below, in the following position, the index finger is flexed. To reproduce this motion, in each frame the angles $\phi_{MC,PP}$, $\phi_{PP,IP}$ and $\phi_{IP,DP}$ are calculated (θ and ψ accordingly).

The basis of a bone represents a rotation matrix, which defines the rotation from the global coordinate system into the current orientation of the bones local coordinate system. Using the basis of an arbitrary bone, for example, bone *A*, at time $t_{i}$ and at time $t_{i+1}$, another rotation matrix can be calculated, which reflects the rotation between time $t_{i}$ and $t_{i+1}$. The matrix $R_{A,0}\left(t_{i}\right)$ represents the rotation from the origin 0 to the bone *A* at the time frame *i*. $R_{0,A}\left(t_{i}\right)$ represents the rotation from the bone *A* to the origin 0 and is equal to ${\left(R_{A,0}\left(t_{i}\right)\right)}^{T}$. With matrix multiplication [23,25], it is possible to calculate the rotation matrix $R_{A}\left(t_{i},t_{i+1}\right)$ from bone *A* between the time frame $t_{i}$ and $t_{i+1}$. From the rotation matrix $R_{A}\left(t_{i},t_{i+1}\right)$ the Cardan angles ϕ, θ, and ψ can be calculated [23,26] and implemented as in the Blender mathutils library [27].

$$\begin{array}{cc} R_{A,0}\left(t_{i}\right) & =\left(\begin{array}{ccc} {\mathrm{xbasis}}_{x} & {\mathrm{ybasis}}_{x} & {\mathrm{zbasis}}_{x}\\ {\mathrm{xbasis}}_{y} & {\mathrm{ybasis}}_{y} & {\mathrm{zbasis}}_{y}\\ {\mathrm{xbasis}}_{z} & {\mathrm{ybasis}}_{z} & {\mathrm{zbasis}}_{z} \end{array}\right) \end{array}$$

$$\begin{array}{cc} R_{A,0}\left(t_{i+1}\right) & =\left(\begin{array}{ccc} {\mathrm{xbasis}}_{x} & {\mathrm{ybasis}}_{x} & {\mathrm{zbasis}}_{x}\\ {\mathrm{xbasis}}_{y} & {\mathrm{ybasis}}_{y} & {\mathrm{zbasis}}_{y}\\ {\mathrm{xbasis}}_{z} & {\mathrm{ybasis}}_{z} & {\mathrm{zbasis}}_{z} \end{array}\right) \end{array}$$

$$\begin{array}{cc} R_{A}\left(t_{i},t_{i+1}\right) & =R_{A,0}\left(t_{i}\right)\cdot R_{0,A}\left(t_{i+1}\right)=R_{A,0}\left(t_{i}\right)\cdot {\left(R_{A,0}\left(t_{i+1}\right)\right)}^{T}\\ \to (\phi ,\theta ,\psi ) & \;\mathrm{calculated}\;\mathrm{from}\;R_{A}\left(t_{i},t_{i+1}\right) \end{array}$$

**Figure 5:**
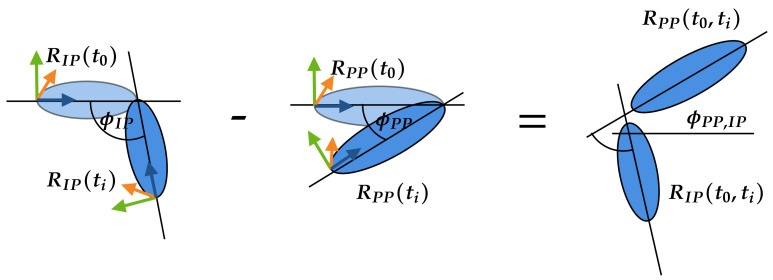
To obtain the angle $\phi_{PP,IP}$ between the proximal phalanges (PP) and intermediate phalanges (IP) the angles $\phi_{IP}$ and $\phi_{PP}$ are needed. Consequently, first the angle $\phi_{IP}$ will be calculated with the rotation matrix from $R_{IP}\left(t_{0}\right)$ at the initial condition ($t_{0}$) and $R_{IP}\left(t_{i}\right)$ at the time step $t_{i}$. Second, the angle $\phi_{PP}$ will be calculated with the rotation matrix from $R_{PP}\left(t_{0}\right)$ at the initial condition ($t_{0}$) and $R_{PP}\left(t_{i}\right)$ at the time step $t_{i}$. The difference between $\phi_{PP}$ and $\phi_{IP}$ defines the required angle $\phi_{PP,IP}$.

To simplify the transfer of the angles to the AMS, the angles of the joints are always calculated in relation to the initial position $t_{0}$ of the hand. The calculation of the Cardan angles is shown by an example in Figure 5. The angle between the proximal phalanges (PP) and intermediate phalanges (IP) of the index finger is calculated. The calculation is divided into two steps. In the first step, the rotation matrix $R(t_{0},t_{i})$ of the IP and PP is calculated at the time $t_{0}$ to the bones IP and PP at the time $t_{i}$. The calculation is made by the basis $R(t_{0})$ and $R(t_{i})$ of the bones and is the same as in Equation (1). To calculate the Cardan angle between the PP and IP, the difference of both rotation matrices $R_{PP}\left(t_{0},t_{i}\right)$ and $R_{IP}\left(t_{0},t_{i}\right)$ must be determined in the same way as in Equation (2).

The calculation of the example as graphically illustrated in Figure 5 is applied to all finger joints.

$$\begin{array}{cc} R_{IP}\left(t_{0}\right) & =\left(\begin{array}{ccc} {\mathrm{xbasis}}_{x} & {\mathrm{ybasis}}_{x} & {\mathrm{zbasis}}_{x}\\ {\mathrm{xbasis}}_{y} & {\mathrm{ybasis}}_{y} & {\mathrm{zbasis}}_{y}\\ {\mathrm{xbasis}}_{z} & {\mathrm{ybasis}}_{z} & {\mathrm{zbasis}}_{z} \end{array}\right)\\ R_{IP}\left(t_{i}\right) & =\left(\begin{array}{ccc} {\mathrm{xbasis}}_{x} & {\mathrm{ybasis}}_{x} & {\mathrm{zbasis}}_{x}\\ {\mathrm{xbasis}}_{y} & {\mathrm{ybasis}}_{y} & {\mathrm{zbasis}}_{y}\\ {\mathrm{xbasis}}_{z} & {\mathrm{ybasis}}_{z} & {\mathrm{zbasis}}_{z} \end{array}\right) \end{array}$$

$$\begin{array}{cc} R_{PP}\left(t_{0}\right) & =\left(\begin{array}{ccc} {\mathrm{xbasis}}_{x} & {\mathrm{ybasis}}_{x} & {\mathrm{zbasis}}_{x}\\ {\mathrm{xbasis}}_{y} & {\mathrm{ybasis}}_{y} & {\mathrm{zbasis}}_{y}\\ {\mathrm{xbasis}}_{z} & {\mathrm{ybasis}}_{z} & {\mathrm{zbasis}}_{z} \end{array}\right)\\ R_{PP}\left(t_{i}\right) & =\left(\begin{array}{ccc} {\mathrm{xbasis}}_{x} & {\mathrm{ybasis}}_{x} & {\mathrm{zbasis}}_{x}\\ {\mathrm{xbasis}}_{y} & {\mathrm{ybasis}}_{y} & {\mathrm{zbasis}}_{y}\\ {\mathrm{xbasis}}_{z} & {\mathrm{ybasis}}_{z} & {\mathrm{zbasis}}_{z} \end{array}\right) \end{array}$$

(1) $$\begin{array}{c} R_{IP}\left(t_{0},t_{i}\right)=R_{IP}\left(t_{0}\right)\cdot {\left(R_{IP}\left(t_{i}\right)\right)}^{T}\;R_{PP}\left(t_{0},t_{i}\right)=R_{PP}\left(t_{0}\right)\cdot {\left(R_{PP}\left(t_{i}\right)\right)}^{T} \end{array}$$

(2) $$\begin{array}{cc} R_{IP,PP}\left(t_{0},t_{i}\right) & =R_{PP}\left(t_{0},t_{i}\right)\cdot {\left(R_{IP}\left(t_{0},t_{i}\right)\right)}^{T}\\ \to (\phi_{PP,IP},\theta_{PP,IP},\psi_{PP,IP}) & \;\mathrm{calculated}\;\mathrm{from}\;R_{IP,PP}\left(t_{0},t_{i}\right) \end{array}$$

#### 2.2.3. Transfer Motion Data to the AMS

**Figure 6:**
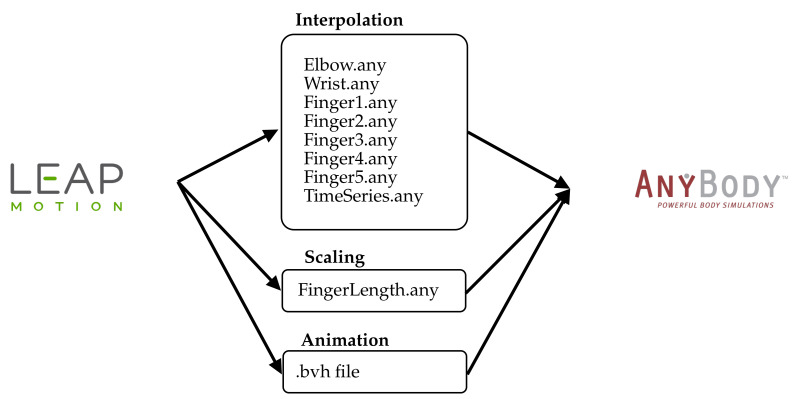
The transfer of data from the LMC to the AMS is divided into three categories. Interpolation: The angles of all joints of the fingers: 1—thumb, 2—index, 3—middle, 4—ring, and 5—pinky, as well as for the wrist and the elbow are stored in vector files. In each file up to three vectors are stored for each joint in order to fully represent the movement in a joint: flexion/extension, abduction/adduction, and rotation. Scaling: The positions of the joints are used to calculate the lengths of the fingers, which are transmitted via the file (FingerLength.any). Animation: The complete movement is stored in a BVH motion file. This serves to restore the recording and visualize the movement. (Source: Leap Motion logo [28] and AnyBody logo [29]).

To transfer the calculated joint angles to the AMS, the angles must be stored in data files. For each finger (1: thumb; 2: index; 3: middle; 4: ring; 5: pinky) as well as for the wrist and the elbow such files are written (Figure 6, Interpolation). In each file, three vectors are stored for each joint to fully represent the movements of a joint: flexion/extension, abduction/adduction, and rotation. along the longitudinal side). In addition, a time series vector is needed for the interpolation. This file TimeSeries.any holds a vector with evenly spaced numbers between zero and one. The length of all vectors are equal to the number or recorded frames. Because the LMC tracks the coordinates of the joints, it is also possible to extract the length of the fingers (Figure 6, Scaling). The solution records and saves the data of the movement as BVH data file and vector files, which are imported into the AMS simulation. Using the BVH file standard allows other tools to edit and view the recorded motion (Figure 6, Animation).

#### 2.2.4. AMS Analysis

**Figure 7:**
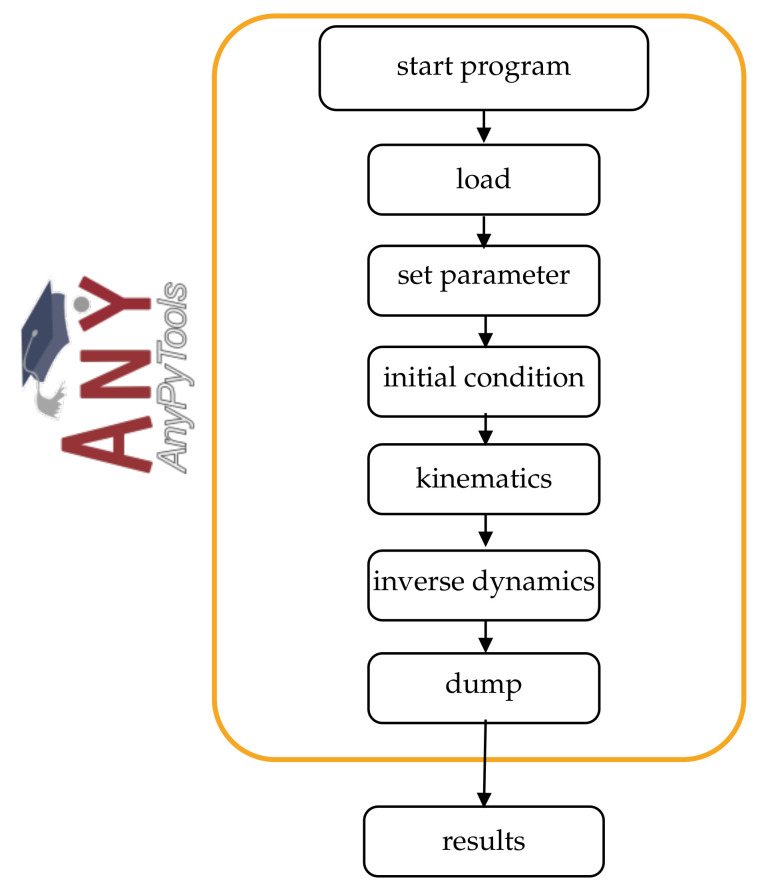
The Python library AnyPyTools can be used to control the AMS. Various actions can be performed using Python and AnPyTool: start the AMS, load the simulation model, set parameter, set the model to its initial conditions, start the kinematic, and inverse dynamics analysis and dumping the result values (source: AnyPyTool logo [30]).

The AMS can be controlled via the Python library AnyPyTool enabling remote controlled simulation and result analysis. The workflow is shown in Figure 7. Depending on the selected operations, each task will be executed automatically. First, the model is loaded (load) and initialized (initial conditions). In addition, parameters to control the model behavior could be set (set parameter). ROSE Motion allows a change in the number of steps for the calculation. The initial position of the model represents an outstretched hand and fingers, by setting all joint angles to zero. Subsequently, the motion (kinematics) is calculated, based on the vector files with the recorded joint angles. In more detail, the joints are driven by a function described in Section 2.1. Finally, the inverse-dynamic problem can be solved. This calculates individual muscle and joint forces. When selected, a dump file of the results is saved to make the data available for further processing and replays.

### 2.3. Validation

**Figure 8:**
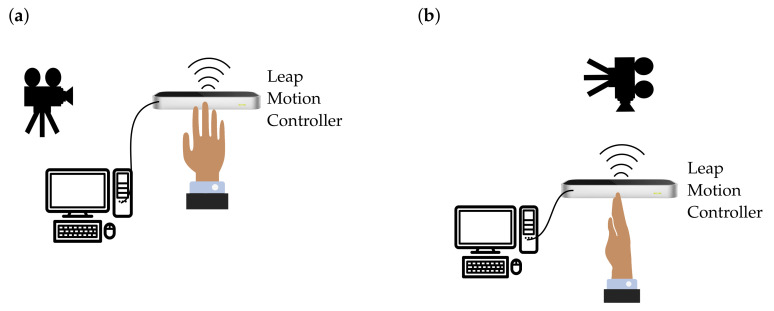
Experimental setup to validate the motion recording of the LMC. (**a**) Experimental setup for a flat hand above the sensor. The position of the camera was on the right to the sensor, to obtain the best view of the index finger making a fist. (**b**) Experimental setup for a vertical hand over the sensor. The position of the camera is above the sensor, to obtain the best view of the index finger making a fist (source: Leap Motion Sensor [28] and icons [32]).

For validation, the motion was captured simultaneously with a Garmin Virb Ultra 30 action video camera and the LMC to evaluate how precisely the LMC records a motion. These two recordings (LMC and video camera) were compared. Each recording started with an outstretched hand and ended with a fist, which was repeated multiple times for comparison. The recording was made at two different shooting positions. In the first shoot, the hand was positioned horizontally above the sensor (Figure 8a). The camera looked at the side of the index finger. Pre-experimental assessments indicated that measurements of the flexion angle of the proximal interphalangeal joint were the most inaccurate. Therefore, the index finger of the proximal interphalangeal joint is used for further analysis.

**Figure 9:**
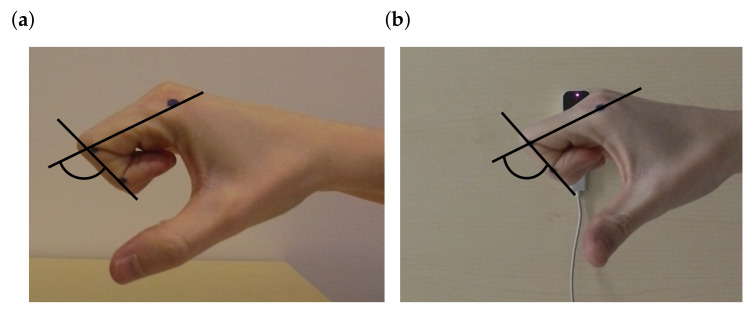
View of the camera on the index finger to track the movement. Black points at the joints of the index finger were tracked by the video tracking software Tracker [31]. With the positions from the tracking software the proximal interphalangeal joint angles (flexion, $\phi_{PP,IP}$) could be calculated. (**a**) Camera on left side of the sensor. (**b**) Camera above the sensor.

The flexion angle ($\phi_{PP,IP}$) was calculated using both recordings, the LMC recording used ROSE Motion and the recording from the Garmin camera. The video analyzing tool Tracker [31] measured the positions of the black markers on each joint of the index finger (Figure 9). The tracking software returned the position of the joints for each frame. From these positions, the angle could be calculated using vectors and angle functions. The same procedure was also used for the vertical hand shoot (Figure 8b). The hand was held vertically above the sensor. To follow the angulation of the hand, the camera was positioned over the sensor. The angle from ROSE Motion and from the camera video were compared accordingly.

## 3. Results

### 3.1. Motion Recording and Simulation with ROSE Motion

**Figure 10:**
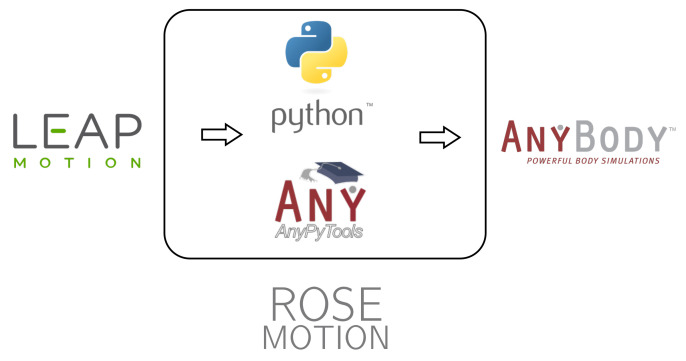
The program ROSE Motion provides an automated interface. The hand movement is recorded with the LMC. With the help of Python and the library AnyPyTools, the movement is transferred to the AMS, which can then be analyzed (source: Leap Motion logo [28], Python logo [33], AnyPyTools logo [30], and AnyBody logo [29]).

The solution ROSE Motion is written in Python and combines useful tools to rapidly integrate the recorded motion in the body simulation. Its main work flow is depicted in Figure 10 and consists of four main features:

- Record
  In the Record function, various settings can be made. It can be specified with how many frames per second the recording is executed. Furthermore, it can be decided whether interpolation files and/or a BVH file is written and its storage location. While recording, a window opens in which the tracked hand movement is visualized in real-time. Upon completion of the recording, an animation to view every frame of the recorded hand is shown.
- AnyBody
  The AMS simulation can be started in the AnyBody feature. Different file sources (including BVH) can be selected, which are modified and copied to the correct location. In addition, one can specify which frames should be included in the simulation (start frame and end frame). Then, all studies to be run by the AMS (initial conditions, kinematic analysis, and inverse-dynamic analysis) can be selected (see Section 2.2.4). Once the simulation has finished, the AMS will be opened and shows a replay of the calculated movement. Further analyses inside the AMS are then directly possible.
- Converter
  In the Convert component, a given BVH file can be converted to the interpolation files used for the AMS.
- Animation
  Opens a BVH file to animate it, a slider can be used to iterate through the frames.

The source code and further instructions are available in [20].

### 3.2. Accuracy Measurement

**Figure 11:**
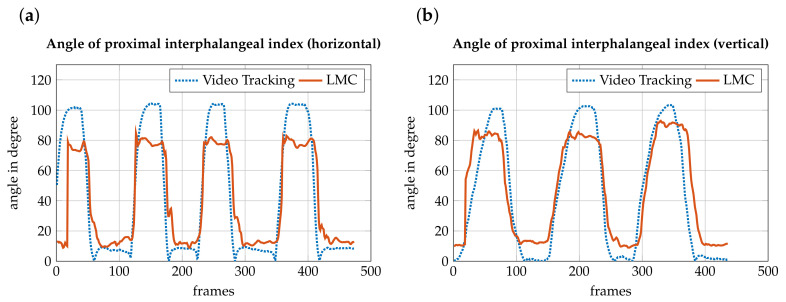
Comparison of the flexion $\phi_{PP,IP}$, angle between the proximal phalanges (PP), and the intermediate phalanges (IP) while forming a fist, recorded by the LMC (red, solid) and the motion tracking of the video camera (blue, dotted). (**a**) The hand is horizontally above the sensor. (**b**) The hand is vertically above the sensor. Because of finger covering, palm covering, and a lack of contrast, the LMC makes a mistake during the recording of a movement in extreme situations (angle > 80 degrees).

To evaluate how precisely the LMC records a motion, the motion was captured simultaneously with an action camera and the LMC. These two recordings were compared. The angle from the video and the recording by the LMC matched. In extreme situations, i.e., the proximal interphalangeal joint angle exceeding 80 degrees, the LMC may not display hand movement correctly. When the fingers are fully extended, the angle should be close to zero degrees. The sensor has a basic angle in the joints, which is assumed to be due to the hand’s basic posture. The difference is shown in Figure 11b. The video has an angle from almost zero degrees, whereas the LMC covers an angle from approximately 10 degrees. Additionally, with a strong buckling, such as a fist, there are major differences. According to video recording, the angle should be approximately 100 degrees, but the sensor cannot detect this extreme bending (Figure 11a). The difference between the recording of the horizontal hand and the vertical hand is the occlusion when shooting. In the horizontal hand, the stretched hand is still relatively well recognized. Strong settlements cannot be distinguished from this perspective. When forming a fist, there are overlaps of finger and hand, causing the LMC to recognize inaccurate values. In the vertical recording, the flat hand is even more difficult to recognize. This is because the sensor can actually only pick up the little finger, while the other fingers are hidden. The inclusion of the fist is better compared to the horizontal recording. The angulation can be better obtained by the perspective. For that reason, the angle is closer to the required 100 degrees.

## 4. Discussion

The aim of this work was to engineer an automatic interface between a LMC and the AMS to capture hand movements and integrate them into an inverse-dynamic musculoskeletal simulation. The LMC provides the positions and orientation of the bones and joints of the recorded hand which can be accessed to calculate joint angles to reproduce the same posture and movement within the AMS using joint drivers.

The publicly available, open source software ROSE Motion fully integrates the LMC and allows integrated hand movement recording and analysis, and editing of the recorded motion. It provides patient specific motion data and finger lengths for the AMS model. The setup, run, and result evaluation of the AMS is directly possible within ROSE Motion. Recorded data can be widely exchanged and used for further applications using the well known BVH format for motion files. Benefits of the proposed solution include rapid motion recording and evaluation of results. The LMC itself is internationally available at low cost and can be used with all major operating systems. No complex experimental setup is required, compared to the time consuming and expensive marker based motion capturing using multiple cameras and complex software. With ROSE Motion it is possible to use the LMC for motion input for musculoskeletal models in biomechnical research.

Inaccuracies can potentially occur either within the AMS hand model (see in [1]) or by LMC recording limitations. The proposed solution is in active development and is currently limited to the integration of the AMS, whereas other inverse-dynamic simulation software as OpenSim [34,35] are out of scope. Although motion export as BVH file might be a solution to work with other tools, a direct integration is preferable. The calculation of joint angles and output to vector files is based on a specific AMS hand model. Updates to the used hand model could introduce breaking changes, requiring fixes of the integration. In addition, a hand model specific neutral-zero position of the arm is used as a reference position for all joint angle calculations. A remapping of coordinate systems might be required if changing the AMS model.

The following limitations correspond to the LMC recorded motion data. The overall functionality of the workflow and integration was proven by the accuracy measurement in Section 2.3. Corresponding limitations because of covering problems—as reported by Rühlicke et al. [14]—were thereby analyzed, proposing an accuracy measurement using video tracking in comparison to the marker-less motion capture recording. Inaccuracy of the anatomical representation of the movement can occur through finger covering, palm covering, or lack of contrast [14]. A similar outcome was discovered in the accuracy measurements comparing the calculated LMC data with tracked video recordings (Section 3.2). The data error was particularly noticeable for the edge cases: rapid movements, forming a strong fist and LMC distant, and covered fingers. In general, it can be said that the LMC provides accurate results for the finger dimensions, palm orientation, and position as well as the orientation of outstretched or slightly flexed fingers.

Inaccuracies could be reduced by various approaches. As Leap Motion evolves, the anatomical presentation and internal hand model will improve. Furthermore, LeapUVC—new interface for Education, Robotics, and more—allows the user to make multiple settings. LeapUVC provides access to the LMC through the industry standard Universal Video Class (UVC) interface. With this application, settings like light-emitting diode brightness, gamma, exposure, gain, and resolution can be changed. The new settings could reduce the error of occlusion [36]. Another approach is the use of two LMCs as in Placidi et al. [37]. The two sensors are positioned in such way that the covering of the hand is minimized. An experimental multiple device support [38] enabling the connection of two LMC devices to a single computer is in active development. The recordings of two sensors could be synchronized and compared. In a future release, the confidence value might allow a statement on how well the internal hand model fits the observed data. The sensor with the better fit should then be selected for each frame to extract the hand data. Consequently, self-coverage, and as a result the error, could be reduced. The herein presented tool can easily be adjusted to benefit from the enhancements in terms of using multiple LMCs.

## 5. Conclusions

An automatic interface between an LMC and the AMS to capture hand movements and integrate them into an inverse-dynamic musculoskeletal simulation was established. This linking shows a fast and low-priced alternative to track forearm motions in biomechanical applications as musculoskeletal simulation models. Limitations because of covering problems were analyzed, proposing to measure the accuracy by using video tracking in comparison to the marker-less motion capture recording. The ROSE Motion software framework is currently limited to the integration of the LMC into the AMS simulation. The approach and software code can be further adapted to be used for additional research cases. Developers and scientists are welcome to contribute, improve, or report issues. The software’s source code is publicly available at [20] and released as open source software under the Massachusetts Institute of Technology license.

## Data Availability

The source code and further instructions are available in [20].

## Abbreviations

The following abbreviations are used in this manuscript:

AMS	AnyBody™ Modeling System
BVH	Biovision Hierarchy
C3D	Three-Dimensional Time-Sequence Data
DP	Distal Phalanges
IP	Intermediate Phalanges
LMC	Leap Motion Controller
MC	Metacarpals
MDPI	Multidisciplinary Digital Publishing Institute
MoCap	Motion Capture
PP	Proximal Phalanges
UVC	Universal Video Class
